# Effectiveness of Simulated Interventions in Reducing the Estimated Prevalence of *Salmonella* in UK Pig Herds

**DOI:** 10.1371/journal.pone.0066054

**Published:** 2013-06-28

**Authors:** Alexander D. C. Berriman, Damian Clancy, Helen E. Clough, Derek Armstrong, Robert M. Christley

**Affiliations:** 1 Institute of Infection and Global Health, Department of Epidemiology and Population Health, University of Liverpool, Neston, Cheshire, United Kingdom; 2 Department of Mathematical Sciences, University of Liverpool, Liverpool, United Kingdom; 3 BPEX, Stoneleigh Park, Warwickshire, United Kingdom; McGill University, Canada

## Abstract

*Salmonella* spp are a major foodborne zoonotic cause of human illness. Consumption of pork products is believed to be a major source of human salmonellosis and *Salmonella* control throughout the food-chain is recommended. A number of on-farm interventions have been proposed, and some have been implemented in order to try to achieve *Salmonella* control. In this study we utilize previously developed models describing *Salmonella* dynamics to investigate the potential effects of a range of these on-farm interventions. As the models indicated that the number of bacteria shed in the faeces of an infectious animal was a key factor, interventions applied within a high-shedding scenario were also analysed. From simulation of the model, the probability of infection after *Salmonella* exposure was found to be a key driver of *Salmonella* transmission. The model also highlighted that minimising physiological stress can have a large effect but only when shedding levels are not excessive. When shedding was high, weekly cleaning and disinfection was not effective in *Salmonella* control. However it is possible that cleaning may have an effect if conducted more often. Furthermore, separating infectious animals, shedding bacteria at a high rate, from the rest of the population was found to be able to minimise the spread of *Salmonella*.

## Introduction

*Salmonella* species are a major cause of zoonotic disease and *Salmonella* spp can be found in many products intended for human consumption, for example eggs, poultry and pork. Consequently, *Salmonella* control at the point of production (i.e. on-farm) is considered important. Although there are more than 2,500 different *Salmonella* serovars [1], *S.* Typhimurium continues to be the most commonly isolated serovar in pigs in the United Kingdom (UK), which has remained the case for a number of years [2], [3]. An abattoir study in 2003 showed that 23.4% (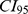: 19.9–27.3%) of pigs were *Salmonella* positive [4]. Although *Salmonella* prevalence differed between regions (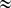
19%–30% [4]), an average prevalence of 23.4% could be representative for the UK. Whilst *Salmonella* infection on-farm might be high, procedures in place in the abattoir (such as scalding, singeing and polishing) can considerably reduce *Salmonella* prevalence, resulting in a low carcase prevalence. However, the bacteria can still survive during these processes [5]–[7] and the resulting carcase prevalence can vary depending on abattoir [2]. Consequently, pork and pork products are considered to be a principal source of human food-borne infections [8]. In the UK 10,071 confirmed cases of human salmonellosis were reported in 2009 [9]; however, the true number of cases is unknown. It is unclear how many cases are directly a result of pork and pork products; in Denmark, domestic pork was estimated to have caused between 3.7%–11.2% of human salmonellosis cases in 2011 [10]. In order to minimise the risk to humans, efforts can be made to reduce the prevalence of infection within commercial herds. Mathematical models can be used to investigate mechanisms that drive *Salmonella* transmission, and can be used to help inform decision making. Within the pig's life cycle, it is the finishing stage which poses the biggest risk to public health. As such, analysing interventions imposed during this stage is of particular interest.

In Berriman et al. [11], models of *Salmonella* transmission around varying structures of British pig grower-finisher farms have been developed and analysed. These models account for the varying flooring types used within the UK, whereby some are ‘solid floored units’ and some are ‘slatted floored units.’ The *Salmonella* dynamics on each type of unit are potentially quite different. For the slatted unit, the basic reproduction number 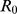 was calculated, being defined to be the average number of secondary infections produced when one infected individual is introduced into a naïve host population (for example, [12]). By computing the value of 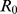, key drivers of *Salmonella* transmission could be investigated. Note that although 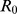 is a useful tool and is widely used in epidemiology, its calculation is insufficient in order to determine whether there is a food risk, since this is dependent on the time frame of infection. This is highlighted through the development of a simple deterministic model in [11]; with base parameter values (Table 1) 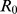 was found to be 0.8204, below the threshold value of 1, but nevertheless infection was found to persist over the time frame relevant to the finishing stage of production. It is possible that the presence of carrying animals keeps the infection sustained for a long period of time. Furthermore, as a number of infectious pigs enter the system, there are a large number of potential infections, which could be sufficient to sustain the infection, despite the low 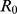 value.

**Table 1:** Definitions of the parameters used in the model.

Definition (units)	Parameter	Estimate	Reference
Number of pigs per pen	*N*	25	[41]
Number of pens on either side of a corridor	*PensPerSide*	20	[41]
Infection rate	*β*	Assume 1.67×10^−3^	–
The rate at which a pig ceases to remain infectious (day^−1^)	*γ*	1/26 = 0.03846	[14], [19]
The rate at which a carrier becomes re-infectious (day^−1^)	*δ*	1/108 = 0.00926	–
The rate at which a pig ceases to carry the bacteria (day^−1^)	*ε*	1/60 = 0.01667	[14]
Loss of immunity (day^−1^)	*ν*	0.5	–
Shedding rate (cfu day^−1^)	*λ*	2.25×10^4^	[42], [43]
Proportion of cfu present ingested (day^−1^): Slatted	*κ*	4.23×10^−4^	[43], [44]
Solid		3.17×10^−5^	
Bacteria death rate (day^−1^)	*l*	1/84 = 0.01190	[45]
Probability of infection from bacterial consumption	*p*	2.30×10^−6^	[19], [46]
Cross infection rate	*α*	Assume 1.14×10^−6^	–
Proportion of faeces that remains in a room	*π*	0.4	–
Proportion of faeces that remains present after cleaning	*q*	0.1	–
Time spent in unit (days)	*T_max_*	108	[26]
Airborne infection rate	*ω*	Assume 1.02×10^−14^	–

Reproduced from Table 2 of [11].

This paper aims to use simulation results of interventions imposed on these models in order to propose practical interventions that could result in on farm *Salmonella* control. By investigating the mechanisms that drive *Salmonella* transmission, information that can inform the development of control strategies can be generated. Furthermore, potential key drivers of *Salmonella* spread are identified. Clearly, any interventions that are tested within the model must be possible to implement in practice to have any value. As such, any modification to parameters applied within the analysis are within a range that is consistent with findings within the literature. Biosecurity (measures and protocols taken to reduce the risk of disease spread on farms [13]) is becoming increasingly important as a means to reduce the risk of a disease outbreak. Biosecurity encompasses a number of aspects (people, cleaning and equipment, for example). The incorporation of cleaning and disinfection within the model of a solid floored unit enables the testing of the effect of cleaning on farm. Physiological stress (such as manure overflow, mixing/moving of pigs and bad feed [5], [14]) is potentially an important factor in *Salmonella* spread as it can cause infected animals (carriers) to resume shedding the bacteria and therefore requires further investigation. The probability of becoming infected after exposure relates to transmission via the faecal-oral route, which is thought to be one of the key routes of transmission and is therefore an important parameter to analyse. Model simulations showed the amount of bacteria shed by an infectious pig was potentially a key driver of *Salmonella* transmission [11]. Further analysis of this result highlights possible interventions that could have an effect on *Salmonella* prevalence.

## Methods

We have previously developed models describing *Salmonella* transmission around two types of British pig grower-finisher farm [11]. The most typical farm structures used within the UK are ‘slatted units’ and ‘solid units,’ the difference being the type of flooring, whereby slatted flooring results in the majority of faeces falling through the slats. Each model enables the testing of a number of possible on farm interventions. Within the slatted model, we were able to compute the basic reproduction number 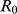, which was calculated by analysing the next generation matrix, as described by [15], [16]. The elements of this matrix consist of the expected number of secondary infections due to a single primary infection in a fully susceptible population, calculated class by class [17]. The value of 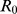 is then given by the dominant eigenvalue of the next generation matrix [15]. Detailed calculations of 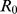 can be found in Appendix 2 of [11].

Within both models, there are a total of 40 pens, each with 25 pigs per pen. Within the slatted unit, the unit is assumed to be divided into 4 rooms with 10 pens in each room (5 pens on either side of a corridor). Within the solid unit however, one large room is used, reflecting realistic practice. Although pigs can shed the bacteria within their faeces in varying amounts [14], [18], [19], pigs are often subclinical carriers of *Salmonella*, which consequently results in the presence of a carrier state [20], [21]. This state can be defined as a pig that is infected with the bacteria in some form, within the tonsils or ileocolic lymph node for example [20], but not shedding bacteria in its faeces. As such, pigs are classed as susceptible (S), infectious (I) (and therefore excreting the bacteria), carrying (C) and recovered (R) within the models [11]. Although both infectious and carrying animals are deemed to be infected (and thus contribute to final prevalence), it is assumed that only infectious animals are capable of passing on infection.

**Figure 1:**
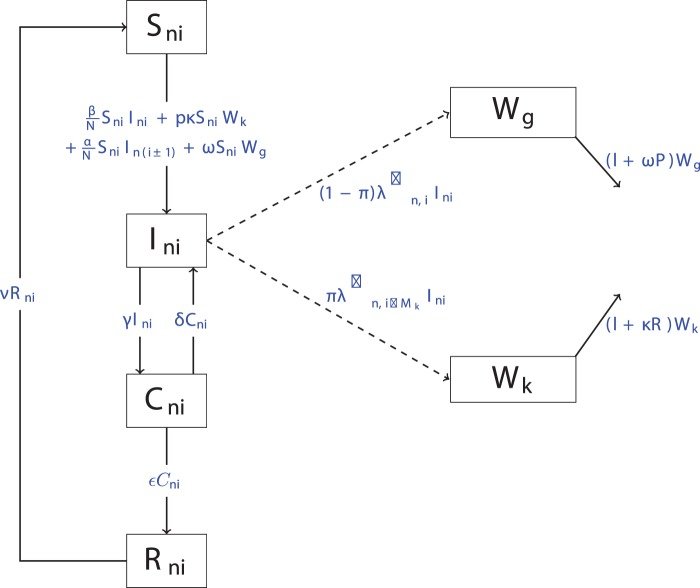
Flow diagram representing transmission routes and other processes within the slatted model. Note: 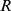 denotes the number of pigs within a room, 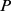 denotes the number of pigs on farm and 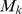 denotes the set of pens within room 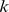. Figure reproduced from Figure 1 of [11].

Due to the different time scales within the model (i.e. between infections and bacterial evolution), the dynamics of the bacteria are treated deterministically in simulation. Thus, a continuous-time, semi-stochastic model is used, which differs from other studies which adopt a discrete time modelling approach [22]–[25]. A flow diagram representing the various types of transmission routes is shown in Figure 1, for a slatted unit. For the solid unit, only one bacterial environment is required; hence the corresponding flow diagram is not shown. The solid unit model also incorporates weekly cleaning and disinfection. Base parameter values are given in Table 1. The models incorporate direct transmission, which can occur within the same pen and between neighbouring pens via contact between susceptible and infectious pigs. Indirect transmission is also incorporated, via the faecal-oral and airborne routes. Infectious animals shed bacteria into their environment. Within the slatted unit, a proportion of faeces is assumed to fall through the slats and so a limited number of bacteria remain available for consumption; within the solid unit, any bacteria shed remain available. It was assumed that the farms operate on an all-in-all-out basis; that is, pigs enter and leave the unit as a group. Furthermore, the farm is assumed to be an exclusive grower-finisher farm and consequently weaners are assumed to be sourced from elsewhere. As such, weaners (at approximately 35 kg) enter the unit (at time 0) and are grown through to finishing in the same building. The model is run for 108 days which is the average time pigs spend within this stage of production [26]. It was assumed that the farm is emptied in its entirety, as all animals are of the same age group. Consequently, the slaughter age prevalence consists of all animals that are classed as either infectious (I) or carriers (C) at the time of slaughter. Although animal prevalence varies greatly, on average in the UK approximately 17% of weaners entering a unit are infected [27]. As such, within the model, a random 15% of pigs entering the unit are assumed to be infectious, and a further 5% considered carriers. The models were validated using data from the Zoonoses National Control Programme (ZNCP) farm visits and results from a British abattoir study. Both of which found between 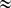
20% to 30% of pigs to be *Salmonella* positive at slaughter, but was on average 23.4% [4]. Unfortunately data were not available for the type of unit pigs came from. As such, this prevalence must be used for both models described.

The model was run for 5,000 simulations in order to present an average end distribution of prevalence. All simulations were run in MATLAB® 7.10 running under Microsoft® Windows® on a desktop personal computer.

## Results

### The effect of cleaning and disinfection

With the incorporation of cleaning and disinfection within the solid-floored unit, the effect of this aspect of biosecurity can be analysed. Cleaning and disinfection is an important aspect of on-farm management practice, especially with regard to biosecurity. Furthermore, it is a highly intensive process and requires a large amount of time to be done efficiently. Within the model, the efficiency of cleaning could be analysed by analysis of the parameter 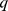; the proportion of faeces that remained present after cleaning. A farm that cleaned to a good standard could remove 90% of the bacteria from the environment for example, compared to a farm with poor cleaning that only removed 10% of the bacteria.

**Figure 2:**
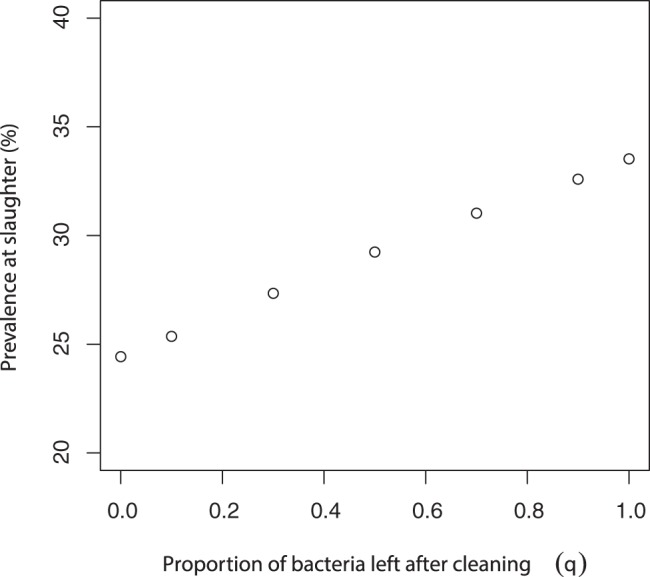
Average slaughter prevalence with varying levels of efficiency of cleaning and disinfection. Results are based on model predictions from the solid unit model. Base parameter value of 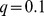.

The model showed that fully effective cleaning alone was not enough to eradicate *Salmonella* once infection was established. However the prevalence (proportion of animals in either infectious or carrying state) was lower if cleaning took place on farm (Figure 2), which implied that cleaning and disinfection was still a worthwhile task. This concurs with a previous study [28] which found that cleaning and disinfection reduces environmental bacteria but fails to eradicate *Salmonella* on farm. Furthermore, an on-farm study of UK pig farms found improved cleaning and disinfection on farm translated into a reduction in prevalence of approximately 10% [29]. The model predicted a reduction in prevalence of approximately 8%, which seemed a relatively good estimate for this effect.

### Proportion of infectious animals entering the unit

**Figure 3:**
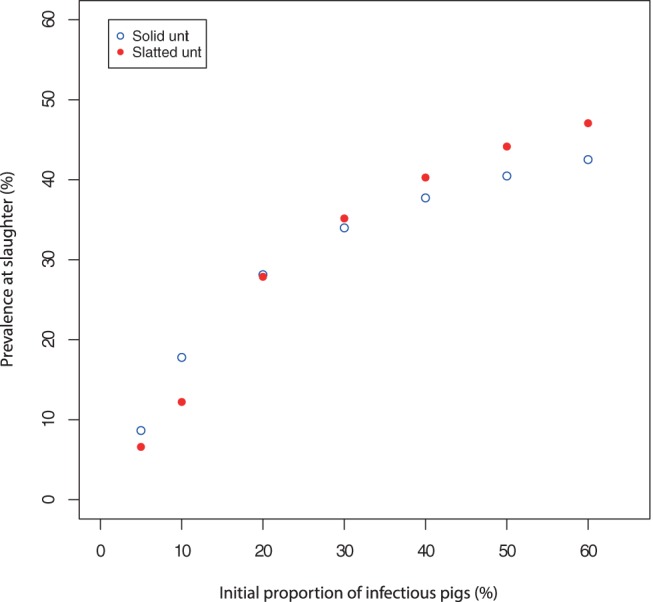
Average slaughter prevalence with varying levels of infectious animals entering the unit. Results are based on model predictions from both the slatted and solid unit models.

*Salmonella* on farm clearly has to be initiated somehow. Within the models presented here, the prevalence at slaughter was dependent on the initial number of infected animals, both infectious and carriers. With the standard number of infected pigs entering the unit (including both infectious (and therefore shedding) and carrier animals, thought to be 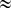
20%), an average prevalence at slaughter of approximately 24.6% and 25.4% within the slatted and solid units, respectively, was found. Within both slaughter age prevalences, the majority of animals were classed as carriers (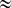
15% with the standard number of infected animals), which can be assumed to be the case for all scenarios unless otherwise stated. With varying levels of infectious pigs entering the unit, it was shown that prevalence just prior to slaughter increased until approximately 60% of pigs entering the unit were infectious (Figure 3), after which any increase in the initial proportion of infectious pigs entering the unit had little effect on prevalence at slaughter and was therefore not included within the Figure. This was true for both the solid and slatted floored models. To analyse how infection is able to spread when animals enter the unit, it is assumed that they are infectious as opposed to carrying, and thus the initial proportion of carriers is 0.

It was also seen that, if low levels of infectious animals enter the unit, *Salmonella* either becomes eradicated (when levels are extremely low; found via simulations with 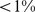 of initially infectious animals) or maintained at low levels. It is important to note that this assumed that no other form of infection exists. As such, this result should be taken on the side of caution, as a low number of infectious animals entering the unit may not be sufficient to ensure a low *Salmonella* prevalence, as external factors may cause additional pigs to become infected.

The prevalence at slaughter was found to be higher within the solid unit until 30% of pigs entering the unit were infectious (Figure 3). For an initial proportion of infectious pigs greater than 30%, prevalence at slaughter is higher in the slatted unit. Within the solid unit, it is possible that cleaning and disinfection on farm is insufficient to eliminate infection from the environment altogether, but can be effective at reducing levels of environmental bacteria when the environment becomes more contaminated. Within both models, at each initial prevalence level, the number of animals carrying the bacteria remained higher than those classed as infectious.

### The effect of physiological stress

**Figure 4:**
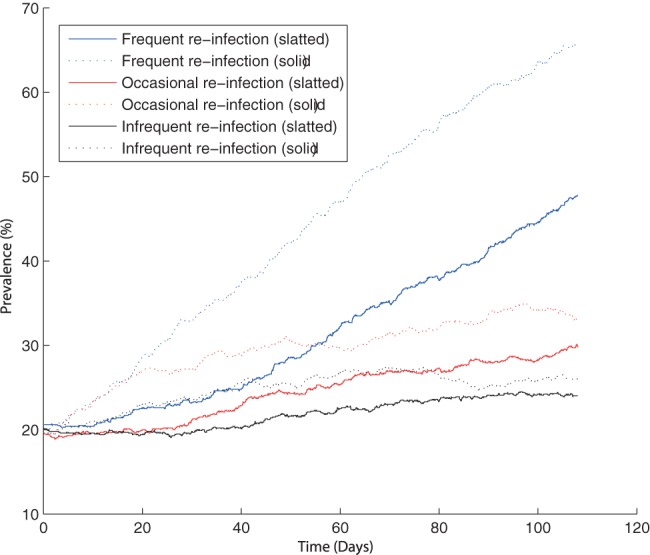
Model predictions showing the effect of the rate of re-infection (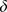) on *Salmonella* prevalence. One typical simulation for each rate and model. Note: 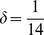 for frequent re-infection, 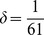 for occasional re-infection and 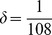 for infrequent re-infection. Base model: infrequent re-infection.

It is well known that any increased stress on pigs that are infected but not shedding (carrier pigs) could cause the animals to resume shedding the bacteria in their faeces [30], [31]. Clearly the majority of stress would be imposed during transport to the abattoir and during lairage, but the general movement of pigs on farm and manure overflow could also have an effect [5], [14]. Within both models, it has been assumed that minimal stress is imposed on the animals on farm (corresponding to infrequent re-infection in Figure 4); increased stress that causes the animals to become re-infectious was shown to influence *Salmonella* prevalence (Figure 4), for each unit structure.

It was shown that the average on-farm prevalence increased as the rate of re-infection became greater (Figure 4). Prevalence at slaughter is higher in the solid unit than the slatted unit whatever the rate of reinfection. However, while this effect is minimal in the base model (infrequent re-infection), the difference become much more marked as the rate of re-infection increases. It was interesting to note that within the solid unit, frequent re-infection consistently appeared to only take effect after approximately 20 days, before which occasional and frequent re-infection showed similar behaviour. By differentiating the *Salmonella* status of the animals with frequent re-infection, it was shown that the numbers of both infectious and carrying animals continually increase. Thus frequent re-infection results in an increase in the number of animals capable of passing on infection, which consequently results in the increase in *Salmonella* prevalence.

**Figure 5:**
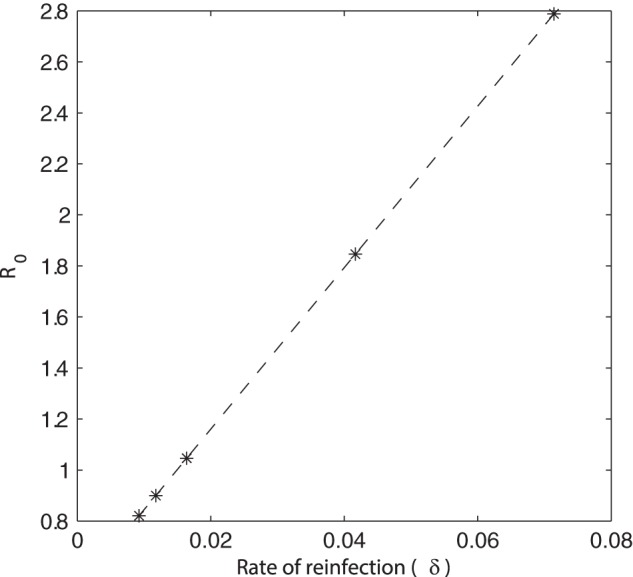
The effect of the rate of re-infection (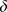) on 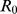 within the slatted unit. Base parameter value of 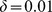.

It is possible that frequent re-infection caused the model to cross a threshold which resulted in the continued increase of prevalence. With the presence of occasional re-infection (after approximately 60 days), the value of the basic reproduction number 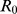, calculated for the slatted model, became greater than 1 (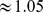) and frequent re-infection caused 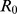 to increase to 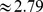 (recall base value 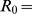 0.8204, Figure 5); this corresponds with the large increase in final prevalence (Figure 4). As such, it is important that stress or any other cause for continual re-infection is minimised in order to keep prevalence as low as possible.

### The effect of the probability of infection

**Figure 6:**
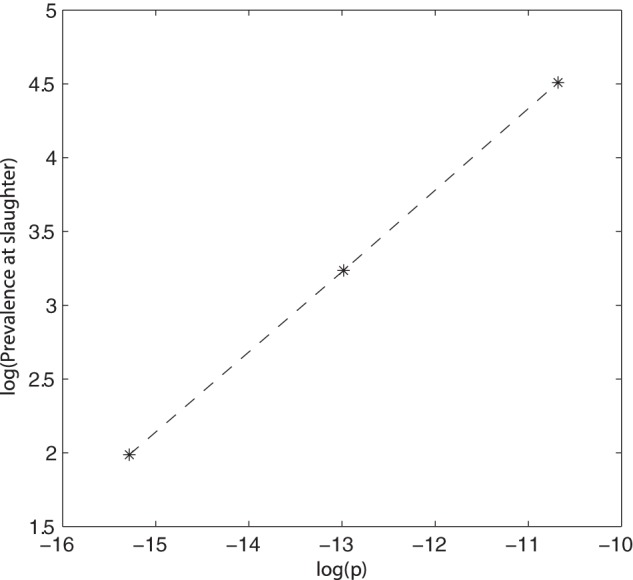
The effect of the probability of infection (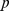) on slaughter prevalence within the slatted unit. Natural logs were used, with base parameter log(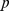) = 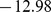. Although these values represent the slatted model, the trend and values are similar to the solid models.

One possible mode of action for *Salmonella* intervention is to reduce the probability of becoming infected after *Salmonella* exposure, which is something that could possibly be achieved via vaccination. With the base probability of infection (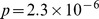), a prevalence at slaughter of 24.6% (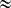
14.4% carriers) and 25.4% (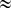
15.4% carriers) for the slatted and solid unit respectively was found. A 10 times reduction in this probability resulted in a reduction in prevalence of a similar magnitude within both models (to 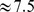
%). Conversely, a 10 times increase in probability resulted in a prevalence of approximately 91% (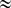
54% carriers, Figure 6). Again this was consistent between both models. As such, it appeared as though, with these levels of shedding, the probability of infection had much the same impact on *Salmonella* prevalence at slaughter regardless of the structure of the unit itself. These results also highlighted a threshold that existed within the model, whereby prevalence is very sensitive to the probability of infection for 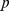 between 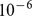 and 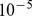, but insensitive outside this range.

**Figure 7:**
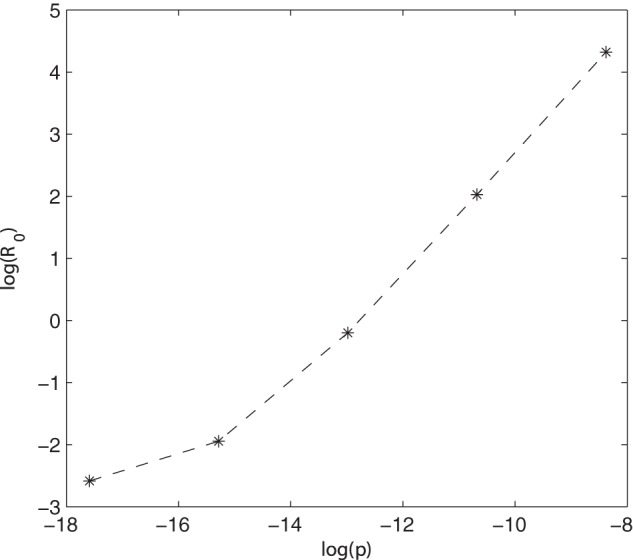
The effect of the probability of infection (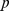) on 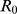 within the slatted unit. Natural logs were used, with base parameter log(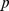) = 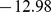.

The probability of infection after *Salmonella* exposure had a major affect on *Salmonella* prevalence; the corresponding effect on the basic reproduction number, 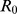, was calculated for the slatted unit. When the probability of infection was decreased 10 times, the corresponding 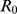 value was 0.1432, compared to the base result where 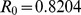. The large increase in prevalence when the probability of infection was 10 times higher was reflected in the 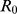 value, increasing to 7.59. This 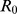 value was greater than 1 and thus a sustained infection would be expected. This increase in 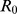 as the value of 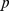 increases is shown in Figure 7.

### The effect of the amount of bacteria shed

Previous analyses of the model highlighted the importance of the shedding rate (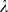) in *Salmonella* spread [11], which concurs with the findings of Lurette et al. [24]. Within both the slatted and solid unit, a ten times higher shedding rate resulted in a slaughter age prevalence of 91.2% and 90.85% (both with 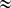
54% of animals classed as carriers) respectively. A potential significant difference between unit structures was highlighted from this analysis, whereby infection within the solid unit was able to spread at a much faster rate.

The finding that a number of pigs shedding high numbers of *Salmonella* in their faeces could have such a drastic effect on prevalence is important. As such, a key issue is to analyse interventions that could have an effect on disease spread even with this high rate of shedding.

#### Effective interventions when shedding is high

From simulation of the solid unit model, it was shown in [11] that with high shedding, weekly cleaning was no longer as effective in *Salmonella* control. In fact, the difference in the average *Salmonella* prevalence between farms with a high level of cleaning and those with a low level of cleaning was less than 1%. This potentially indicates that infection can become established quickly and consequently weekly cleaning of the farm is rendered inadequate. It is therefore quite possible that in order for *Salmonella* control when shedding is high, cleaning must be conducted more often in order to minimise the amount of bacteria that pigs are exposed to. This however would require further investigation.

**Figure 8:**
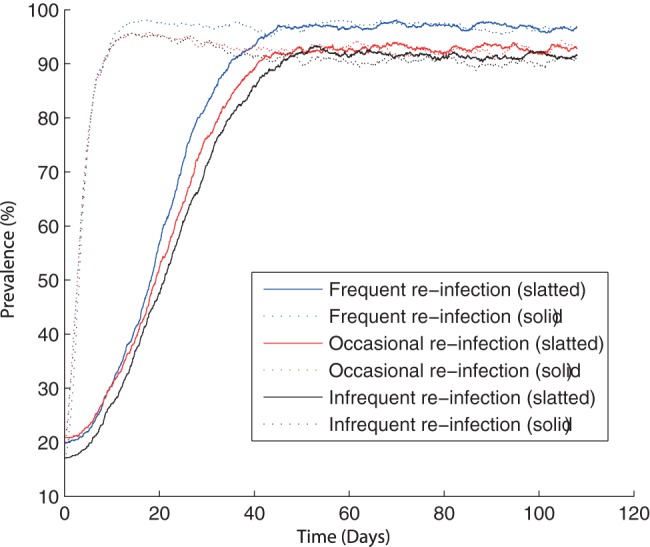
The effect of the rate of re-infection (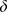) on *Salmonella* prevalence with high shedding. Results are based on model predictions from both the slatted and solid unit models. One typical simulation for each rate and model. Note: 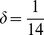 for frequent re-infection, 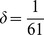 for occasional re-infection and 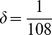 for infrequent re-infection. Base model: infrequent re-infection.

Previously, an increase in the rate of re-infection was shown to have quite a large effect (Figure 4). However, when shedding was higher, the rate of re-infection had a much smaller effect on *Salmonella* prevalence at slaughter (Figure 8). Both models appear to have a very similar slaughter age prevalence, however the dynamics are quite different; the spread of infection was more gradual within the slatted unit. With the presence of higher shedding, the value of 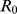 (for the slatted unit) for infrequent re-infection 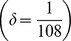 was found to be 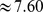 and became progressively higher as the rate of re-infection increased. Although 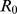 increases, it appears that the value for infrequent re-infection was sufficiently large for any increase to have a minimal affect on prevalence, which was highlighted by simulation (Figure 8).

A point of interest was the extremely small difference in prevalence with these varying rates of re-infection; a difference of approximately 6%. This is clearly a considerable contrast with ‘normal’ shedding levels, where frequent re-infection resulted in a prevalence of approximately 40% higher than infrequent re-infection. When shedding was high, the majority of the population was already infected in some form, shedding or carrying, and frequently changing between states did not have a large effect. However, with ‘normal’ shedding levels, frequent transition between carrying and shedding (and therefore infectious) states can have a drastic affect, by increasing the number of animals that are capable of passing on the infection.

Within both models, the probability of infection was found to be an important parameter when shedding was at ‘normal’ levels. With the higher rate of shedding, a 10 times reduction in the probability of infection saw prevalence within both models fall to approximately 25% (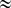
15% carriers), a reduction in prevalence of approximately two thirds. It could be concluded that the probability of becoming infected after *Salmonella* exposure is an important factor in disease spread regardless of the shedding rate, and a potential key driver of *Salmonella* transmission.

**Figure 9:**
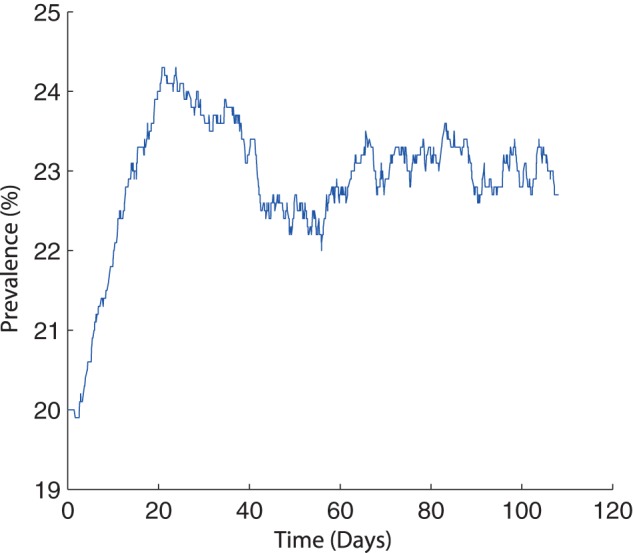
One typical simulation showing the result of containing infectious pigs shedding high numbers of bacteria. Animals are assumed to be shedding the bacteria at a rate of 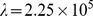 and are contained within 1 room of a slatted unit.

When animals shedding bacteria at a high rate (potentially “super-shedders”) were randomly spread throughout the unit, prevalence at slaughter was approximately 90%. However, if all infectious pigs, shedding at a high rate, were contained within 1 room of the building (whereby infection is able to spread between pens within a room, but is less likely to spread between rooms, Figure 9) then in general, this could be enough to halt transmission as infection was unable to spread throughout the whole unit. Containing all infectious animals to 1 room limited the number of animals that were exposed to the bacteria and consequently limited *Salmonella* transmission. Although farmers cannot easily identify individual pigs that become infected, this finding could still be exploited by attempting to keep pigs in groups of pens with solid divisions, thus ensuring every effort is made to prevent contact between pens and essentially create different epidemiological groups. Clearly it is not easy to identify all infected animals due to many animals being asymptomatic. However, if the assumption that carrying animals are incapable of passing on the infection is true, then the focus should be on containing infectious pigs (i.e. those animals that are shedding the bacteria). As long as stress on farm is minimised (i.e. carrying animals rarely become re-infectious) then the presence of animals carrying the bacteria elsewhere in the unit could have a minimal effect.

## Discussion

We have analysed semi-stochastic transmission models describing the dynamics of *Salmonella* within 2 types of grower-finisher herds, as detailed in [11]. Each model enabled the assessment of different aspects of *Salmonella* dynamics. The slatted floor model allowed the calculation and analysis of the basic reproduction number 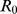, which has not previously been analysed in this context. It was shown that 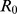 was insufficient in determining whether the disease was eliminated quickly or able to persist. When 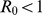, the disease does not necessarily disappear (over the relevant time period). Consequently, 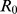 calculations may not be the most effective way of examining interventions applied within this system due to the complex dynamics.

Findings from the solid unit model highlighted some key issues with cleaning and disinfection on-farm. The model found cleaning to have minimal effect on *Salmonella* prevalence when shedding was high, which is likely due to the rate at which infection was able to spread. In an attempt to counteract this high uptake of infection with the use of cleaning, it is possible that more frequent cleaning could minimise *Salmonella* spread. Although this has not been implemented here, the fact that cleaning does have some effect on reducing the prevalence was thought to confirm this supposition. However, further analysis would be needed in order to determine whether this would be feasible, beneficial and economically viable.

Separating animals that shed bacteria at a high rate was shown to have an effect on *Salmonella* prevalence, as the infection was unable to spread throughout the whole unit. Although this is a relatively simple intervention, difficulties arise in its implementation as infectious animals are hard to identify. Any advances in the ability to identify infected animals could result in an increased potential to apply this intervention effectively.

The addition of prebiotics to drinking water has been shown to be associated with a reduction in *S.* Typhimurium shedding [32]. Probiotics on the other hand have been shown to have little effect on shedding, but do show signs of reducing the presence of the bacteria internally (in the mesenteric lymph nodes for example), which implies that probiotics and prebiotics could alter the gut microflora composition to the benefit of the animal [32]. Acidification of feed has been shown to inhibit *Salmonella* growth, which results in a reduction in infection levels and consequently the amount of bacteria shed [33]. The type of food used could also have some impact on the dynamics, for example wet feed has been associated with a reduction in shedding [33]. Clearly there are a number of possible interventions that could be implemented with regard to feed, although a large factor for decision making is cost. In changing the whole system to use wet feed, it is quite possible that a large scale renovation of the unit would need to occur.

A factor that had a major impact on prevalence was the probability of infection after *Salmonella* exposure. A decrease in the probability of infection was found to be extremely influential in *Salmonella* control even when shedding was at high levels. In order for these simulated interventions to be of use, there needs to be a practical way in which such interventions can be implemented. Although it has been shown that adding antibiotics to feed can reduce the amount of *Salmonella* shed by an infected animal [34], with the presence of resistant *Salmonella* strains, the addition of the antibiotic to the resistant *Salmonella* strain can increase the quantity, duration and prevalence of faecal shedding [35]. Consequently, the use of antibiotics is somewhat controversial due to the potential increased risk of generating antimicrobial resistance, and therefore unlikely to be implemented in order to control *Salmonella*.

It would be interesting to see if vaccination would have an effect on two aspects by decreasing the amount of bacteria shed when an animal becomes infected and/or reducing the susceptibility of the animals. Although vaccinations could be useful in helping to prevent clinical salmonellosis in pigs, the capacity for a vaccination to make a contribution to reduce shedding in pigs remains unproven. Various vaccines have been developed (for example Salmoporc (IDT BIOLOGIKA) licensed live vaccine) but are not widely used on farm. It is possible that vaccination is scarcely used due to the potential for the vaccine to interfere with current control programs relying on serology [36]. Vaccination against viral infections is expected to limit the chance of bacterial infections [37] and should aim to prevent colonisation of the host and minimise the shedding of the pathogen [38]. A number of studies have been conducted that show vaccination is associated with a reduction in isolation of *Salmonella* in slaughter weight pigs with a reduction of clinical symptoms and colonization of the animal [36], [39], [40]. Vaccination may have a role where *Salmonella* prevalence is high, whereby piglets from vaccinated sows with high antibodies should have high maternal antibody levels. Furthermore, vaccination of sows would spread the cost of vaccination over all piglets. However, this would provide no protection during the later stages, once maternal antibodies had waned.

Physiological stress is a known factor in reactivating *Salmonella* shedding [30], [31], however it is possible that carriers of the bacteria can resume shedding intermittently without any physiological stress. Within the base model, it has been assumed that animals resume shedding infrequently, which is thought to be a fair assumption, assuming best management practices and high animal welfare standards. However, changes in the rate of re-infection were shown to have a large impact on the slaughter age prevalence. Consequently, the impact of carrier pigs in the *Salmonella* transmission process may be high. As such, a future direction of this research could be to analyse the effect of carriers on the dynamics. For example, testing different proportions of infectious and carrier animals entering the unit could change the dynamics.

Both models (slatted and solid units) exhibited similar behaviour with regard to *Salmonella* prevalence at slaughter age with a number of scenarios, such as changes in the shedding rate and the probability of infection. There were nevertheless implications with regard to the application of an intervention. With the accelerated uptake of infection within the solid unit, the time at which an intervention should be applied in order to be as effective as possible may need to be during the initial uptake of infection. However, this would require further investigation.

Some issues may arise however with implementing certain interventions. For example, biosecurity practices and standards vary across the country (and elsewhere), thus biosecurity interventions will be associated with biosecurity policies within each location. Furthermore, it is possible that regional or national level actions could impact the infection of post-weaning pigs. Although the interventions described in this paper focus on pre-harvest measures to reduce *Salmonella* burden, there are other procedures that could be utilised. For example, there may be added value in developing a surveillance programme that could identify positive breeder units from negative units. This in turn could be an effective way of sourcing piglets from negative units and, in the long run, be more cost effective.

In conclusion, our study found the probability of infection after *Salmonella* exposure and the number of bacteria shed by an infectious animal to be key drivers of *Salmonella* transmission. These results should help inform the future direction of research regarding *Salmonella* transmission in pigs. Further research is required in order to identify possible measures that could be quickly and efficiently implemented to control these factors. The development of effective vaccinations or improved biosecurity measures (such as improved management practices and efficient control of sick animals) for example, may have the desired effect. Minimising physiological stress can also result in a reduction in on farm *Salmonella* prevalence. An intervention focusing on this aspect, by effective management and biosecurity practices for example, could be more realistic and easier to implement in the short term, and still provide worthwhile results.
